# Supplementary material for: An Interactive Approach to Teaching the Clinical Applications of Autonomy and Justice in the Context of Discharge Decision-Making
Source: MedEdPORTAL. 2020 Oct 16;16:10992. doi: 10.15766/mep_2374-8265.10992 (PMC7566224; doi:10.15766/mep_2374-8265.10992)
Supplement: Supplementary file 1 — Facilitator Guide.docxInstructions for Creating Interactive Table.docxStudent Handout.docxPretest.docxPosttest and Feedback Form.docx [file mep_2374-8265.10992-s001.zip › A. Facilitator Guide.docx]

**Healthcare Decision-Making in the Context of Stroke**

**Educational Goals:**

By the end of the activity, students will be able to answer the following:

1. How does stroke affect competence?
2. Who can serve as a surrogate decision-maker for a patient judged legally incompetent?
3. What are the different levels of rehabilitative care, and how are recommendations for placement determined?

This activity will also simulate the challenges patients face in directing their own care with the goal of promoting empathy amongst students.

**Summary of the activity:**

- Students will first be split into two teams.
- Each team will be given an arterial territory and asked to identify the deficits, and then the deficit that would most impact their ability to direct their own care. Students will proceed through the activity as if they were limited by that deficit.
- The facilitator will then lead students through 4 days of a post-stroke hospital stay
  - Each day, they will confront a “task” based on choices patients face during their hospital stay. Each task will lead into discussion questions related to an educational goal (listed above)
  - Students will proceed through the activity as if they were the patients, with the goal of being discharged to a facility that is appropriate for both their neurological needs and personal values.

**Materials:**

- Facilitator Guide
- Pre-activity and post-activity assessment
- Student Handout
- Table Entry Cards

**Facilitator Guide**

**Facilitator Guide Key**

- Red Text is meant to direct you towards your next instructions as facilitator

**Facilitator Guide Key**

- Red Text is meant to direct you towards your next instructions as facilitator
- Blue checkboxes such as this one are meant for you to use to keep track of your position in the guide. They are next to each sub-segment of the activity you must facilitate
- **Blue Text** indicates a discussion question you must ask students

**Pre-Assessment**

Hand out the assessment. Give students 5-7 minutes to complete the assessment.

**Introduction to the Activity**

Facilitator:

- 1) Read the learning objectives on page 1 aloud for the students.
- 2) Read what’s below aloud to set up the situation for the simulation:
  - In this activity, you’re a 68 year old male patient, a retired novelist, with a past medical history of hypertension and diabetes. When your wife returns from an all-day golf trip, she finds you collapsed in the kitchen and unable to get up. She calls 911. In the ED, CT scan shows a large evolving infarct. Your doctors determine you are not eligible for treatment, and you are admitted to the hospital.
  - According to the American Stroke Association, the average length of hospital stay is 4 days after an ischemic stroke.^1^ This activity is meant to simulate the challenges stroke patients face during the course of their hospital stay. You will go through 4 “days” of this simulation.
  - First, you will be split into two teams, and each team will be given a different arterial location where the stroke occurred.

**Day 1—Determining competence (10 min)**

- Facilitator:
  - Read aloud: this is the start of “Day 1” of the activity, and that the goal for this day will be to explore the concept of competence.
  - Split the group of students into two teams.
  - Assign one of the following territories to each team of students:
    - Left MCA (aphasia)
    - ACA (executive functioning/changes to personality)
- Ask each team of students:
  - **1) What deficits would result from occlusion of their assigned arterial territory?** (30 second discussion time in teams, then share as a large group)
    - Review with students the different arterial territories if necessary
    - Answer:
      - MCA: deficits would include loss of language function, some motor and sensory deficits as well
      - ACA: loss of executive function, some personality changes
        - **How might you assess if a frontal lobe injury affects decision-making?**

Answer: gather collateral from family to assess baseline

- - - - - **How might you assess judgement in clinic, if family were unavailable?**

Answer: See if a patient gives an inappropriate response to a common-sense question; for example—what would you do if you were home and smelled smoke?

- - **2) Are patients with these deficits legally competent?** (30 second discussion time in teams. Ask the follow up questions below as appropriate. Afterwards, share thoughts as a large group)
    - Students should respond by stating that competence is a legal definition, and cannot be determined by a physician. The question above is meant to elicit any misconceptions; use the discussion questions below to clarify if misconceptions do arise.
    - **What is the difference between capacity and competence?**
      - Capacity is a medical determination, made by physicians
      - Competence is a legal determination, made by a judge
    - **What are the requirements to determine capacity?**
      - Suggested Answer:
        - Capacity is a specific sub-component of cognition related to the patient’s ability carry out all that is necessary to maintain their health.^4^

They need not be functionally capable of doing all that is necessary for themselves, but they must be capable of recognizing where they need assistance and making arrangements to have others assist them where needed.^4^

- - - **What are some ways you can promote patient autonomy for patients with limited capacity?**

**Day 2 –Hierarchy of surrogate decision-makers (15 min)**

- Facilitator:
  - Read aloud: it is now “Day 2” of the activity. The focus of this “day” will be on contacting the patient’s families and surrogates for decision-making.
- Contacting patient families
  - **Given the deficit assigned to your team, how might that deficit limit the patient’s ability to tell their providers which family members they would like informed of their situation? What are some ways to work around those deficits?** (1 min discussion in teams, then share as large group)
- Next, give an update (read aloud the text below) to the patient situation:
  - After your first day in the hospital, your doctors establish that your situation is severe enough that you will need a PEG tube placed. This is a procedure that requires consent, and you are tasked with finding out who can give consent on behalf of the patient.
  - Read aloud:
    - The patient comes from a large family: he has his wife, age 66. Two adult children, aged 31 and 28. He has one living parent, his mother, who lives nearby. He has three cousins who are also in-state, and are very close to him. He also has one sister, who lives out-of-state in California, who visits when she can.
  - **Whom must the physician contact in order to obtain consent for this procedure?** (30 second discussion time in teams. Afterwards, share thoughts as a large group)
- Explain the hierarchy below to the students. It is reproduced in the student handout, which will be given to them later.

Spouse

Adult Child

Parents

Adult Sibling

Nearest Adult Relative

**Figure 1**: Hierarchy for surrogates in Ohio. Information obtained from the American Bar association^9^. Resources specific to each state can be found at https://www.americanbar.org/content/dam/aba/administrative/law_aging/2014_default_surrogate_consent_statutes.authcheckdam.pdf.

**How does this hierarchy help you as the provider promote patient autonomy?**

Read aloud: While the hierarchy above only applies explicitly to withdrawal of care, this is also the only hierarchy explicitly available, and is thus extrapolated to other decisions requiring a surrogate as well, including consent for a surgical procedure.

- Majority rule prevails for adult children and siblings who disagree and are equal priority surrogates.^2^
- Ethics committees can be convened to make decisions in the absence of an appropriate or immediately available surrogate.

**Are there situations in which adhering to the hierarchy above may not be best representative of the patient’s wishes?**

Suggested Answer: Yes, for example if the patient is closer to their sibling than their child, the hierarchy above may not be best representative of the patient’s wishes.

**Day 3—Post-Hospital rehabilitative care (15 min)**

- Read aloud: It is now “Day 3” of the activity. The focus of this day will be on understanding the different levels of rehabilitative care.
- Give a situation update, read the following aloud:
  - Providers were able to reach the patient’s wife, and she gave consent for the PEG tube. The PEG tube is scheduled for a later date, and she would now like to know more about the different options for rehabilitative care.
- Read aloud: Your goal for the day will be to identify the best option for post-hospital rehabilitation for each of their assigned deficits.
- Ask:
  - **What sorts of long-term care needs will patients have given each team’s assigned deficits?** (30 second discussion time in teams. Afterwards, share thoughts as a large group)
- **Activity**
  - For this day, students will work together as a large group to put together the table below, using the blank felt table provided. At the beginning of the activity, lay out the felt on any available flat surface.
  - Read aloud: You will be given the entries for the table below. Each of you will place an entry where you think it should go using the Velcro on the back. The text color on the cards corresponds to the appropriate column.
  - Shuffle the table entry cards provided. Use the key below to check their work. As the students fill in the table, use any discrepancies to discuss.

|  | **Level of Medical Needs** | **Amount of Therapy Available** | **Frequency of Doctor visits** | **Other requirements** | **Cost** |
| --- | --- | --- | --- | --- | --- |
| **LTAC (long-term acute care facility)** | Severe | NA | Constant ICU- level care | Medical needs such as ventilator dependence, severe decubitus ulcers, etc | $$$$$ |
| **Acute Rehab** | High | 3+ hours of therapy, 5 days a week | 5+ days a week | In-hospital stay, patient must be able to participate in therapy (non-lethargic, attentive) | $$$$ |
| **Subacute Rehab /Skilled Nursing Facility (SNF)** | Medium | 1-2 hours of therapy 5 days a week | As needed, generally 3 times a week | Patient need not be able to attentively participate in rehab | $$$ |
| **In-Home rehab** | Medium-Low | 2-3 hours per week | Scheduled follow-ups | Non-ambulatory, Requires consistent family support | $$ |
| **Outpatient Rehab** | Low | Few hours of therapy 2-3x a week | None | Patients should be ambulatory | $ |

**Table 1**: Comparison of the different levels of rehabilitation following a stroke.^3^

Give the students their student handout

Update each team as to the situation of their patient below.

- Have the MCA team listen to the prompt below and respond to the question first.
  - Read Aloud: Assume the patient is now alert, attentive, and participates in rehab. However, at this point he is not ambulatory.
  - **Which level of care will be appropriate for the patient?**
    - Answer: appropriate level of care for this patient would be Acute Rehab
    - Follow-Up Question: **If the patient were not participating, how would this change discharge planning?**
      - Answer: If the patient is not participating in rehab, they do not qualify for Acute rehab
- Next ask the ACA team the following**:** **The patient is ambulatory, attentive, and sometimes participates with rehab. However, they have lost behavioral control as a result of their stroke. They are pulling out their IVs, and refuse to stay in bed. They have some weakness in their legs but are able to walk with assistance. Although they require supervision, their family is unable to be with them at all times. What is the appropriate level of care?**
  - Answer: SNF, for purposes of supervision

(continues on next page)

- - Ask this follow-up question: **If the patient did not require supervision, what would be the appropriate level of post-hospital rehab care?**
    - Answer: This patient would require outpatient therapy. You would refer them to support groups for stroke survivors if they were interested.
  - Ask this follow-up question to the entire group:
  - **If a patient’s function after a stroke were at or near their original baseline level of function, would they need rehabilitation?**
    - Answer: No, they would not need rehab

**Do you feel that these guidelines help promote the ethical principle of justice in resource allocation?**

**Day 4 –Final planning for discharge (5 min)**

- Give the following situation update (read aloud the text below):
  - Doctors have decided that the patient is ready for discharge: the MCA patient to Acute Rehab, and ACA patient to a SNF
  - Thought the MCA was referred for acute rehab, insurance denied it and he’s going to SNF
- Read aloud: We have reached the final day of the simulation, Our task for the day is discharge, which really means answering any questions you may have. Before we go into questions:
  - **Optional: Inform students that medicare.gov has additional resources to help patients directly compare inpatient rehab facilities.**
    - [**https://www.medicare.gov/inpatientrehabilitationfacilitycompare/**](https://www.medicare.gov/inpatientrehabilitationfacilitycompare/)
    - **This link is on the student handouts, and is a national resource.**
- Use this time to finish discussion on any topics that came out of the activity but were unable to be addressed completely.

**Post-Activity Assessment: (10 min)**

1. Give students the post-activity assessment and feedback form to fill out
