# Supplementary material for: An Interactive Approach to Teaching the Clinical Applications of Autonomy and Justice in the Context of Discharge Decision-Making
Source: MedEdPORTAL. 2020 Oct 16;16:10992. doi: 10.15766/mep_2374-8265.10992 (PMC7566224; doi:10.15766/mep_2374-8265.10992)
Supplement: Supplementary file 1 — Facilitator Guide.docxInstructions for Creating Interactive Table.docxStudent Handout.docxPretest.docxPosttest and Feedback Form.docx [file mep_2374-8265.10992-s001.zip › B. Instructions for Creating Interactive Table.docx]

**Instructions for Creating and Using the Interactive Table**

Please follow the instructions below to create the interactive table students use in simulated “Day 3” of the activity. This document also contains a mock-up of how to use the table to facilitate “Day 3” of the activity.

**Suggested Materials:**

- Felt of any color, 24 in x 36 in
- Printouts of cards (Attached)
- Velcro with adhesive ability
- Index cards, at least 3” x 5” (35)
- Glue
- Scissors

**Instructions:**

1. Print out attached cards below. Cut out cards using the dotted lines (3” length; 2.5” height)
2. Glue each cutout to an individual index card. Trim the index cards to fit size of printed out cards.
3. Cut the Velcro into small segments such that they fit on the backs of each index card. Separate the two sides of the Velcro (loop side and hook side). Use the Velcro adhesive to attach the loop side of the Velcro to the back of each index card.
4. Space cards out evenly on the felt as shown in the Facilitator Guide. Use the adhesive on the Velcro to attach the hook side of the Velcro to the spaces where each card should go.
5. Use the Velcro the affix cards to their final locations on the felt.

**Figure 1: Mock-up of interactive activity students complete on simulated “Day 3”**

a. b.


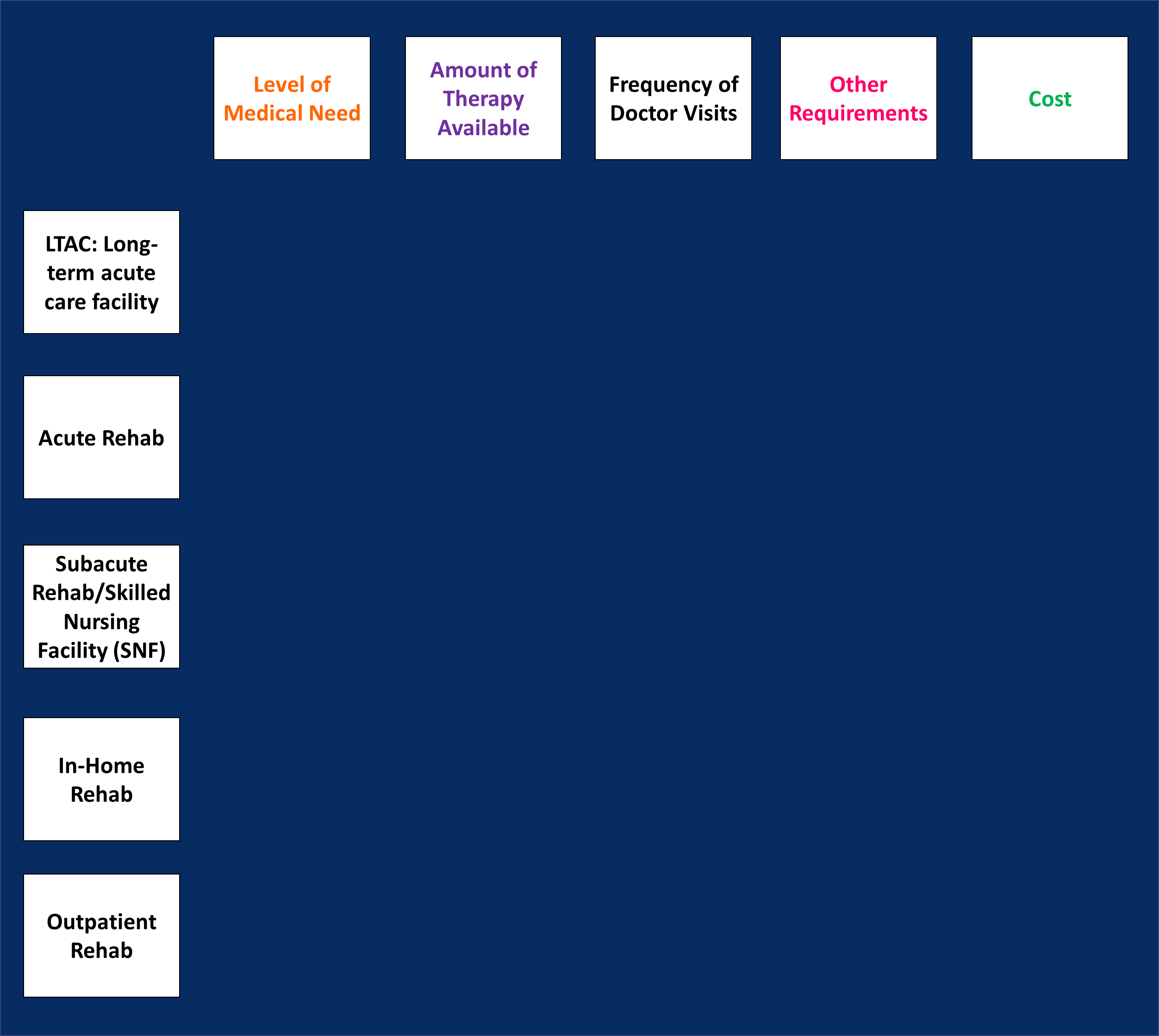

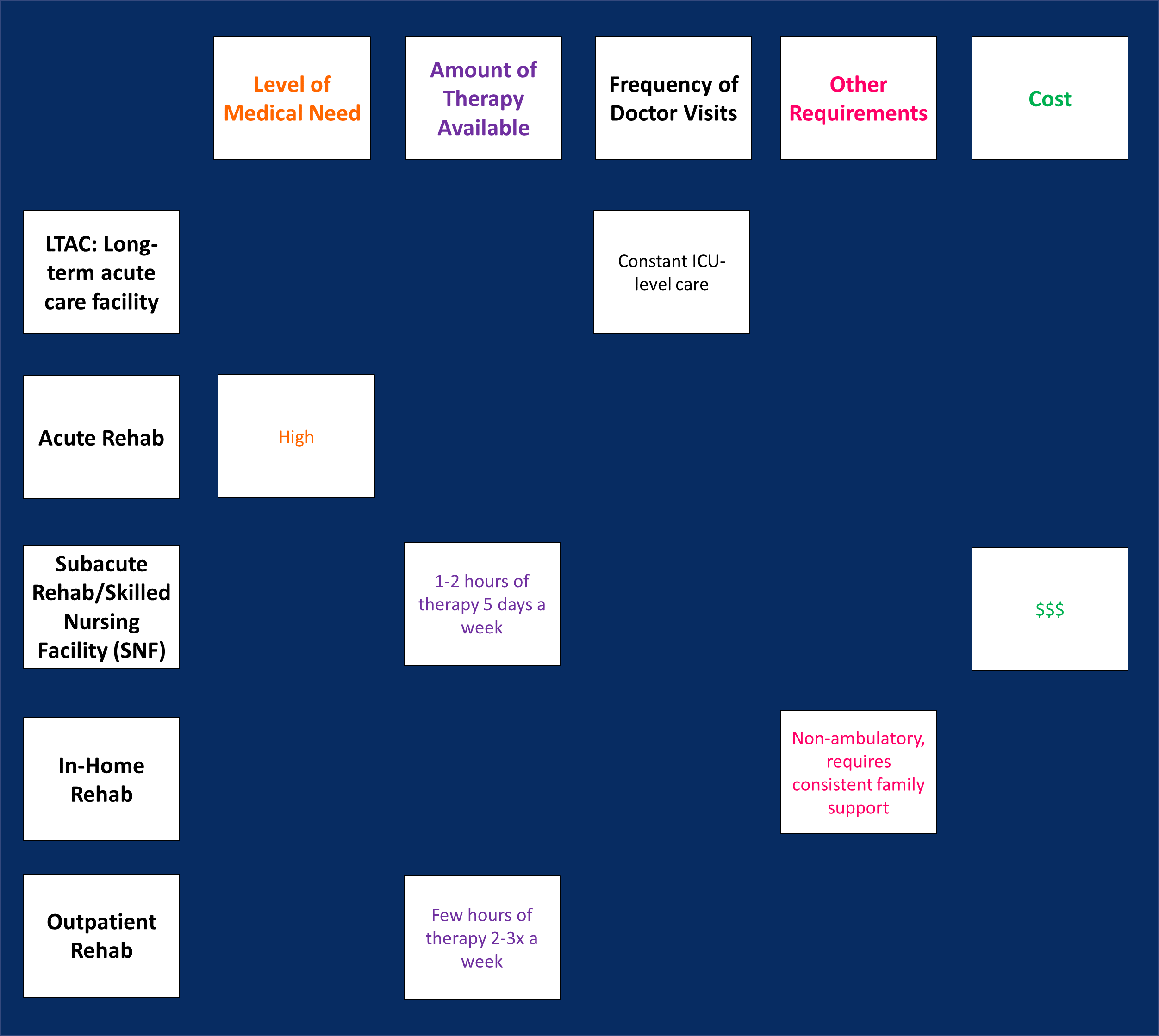


c. d.


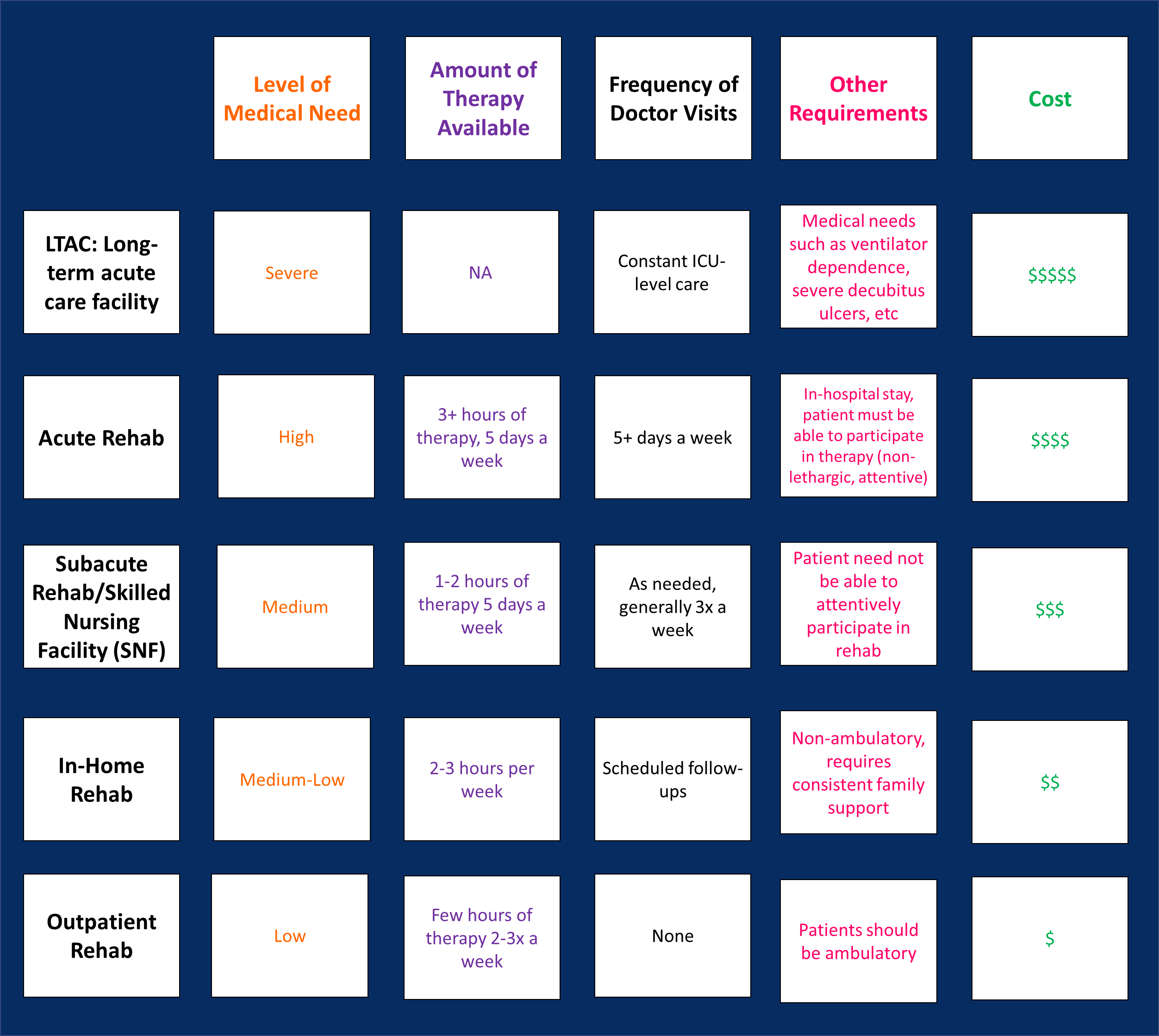

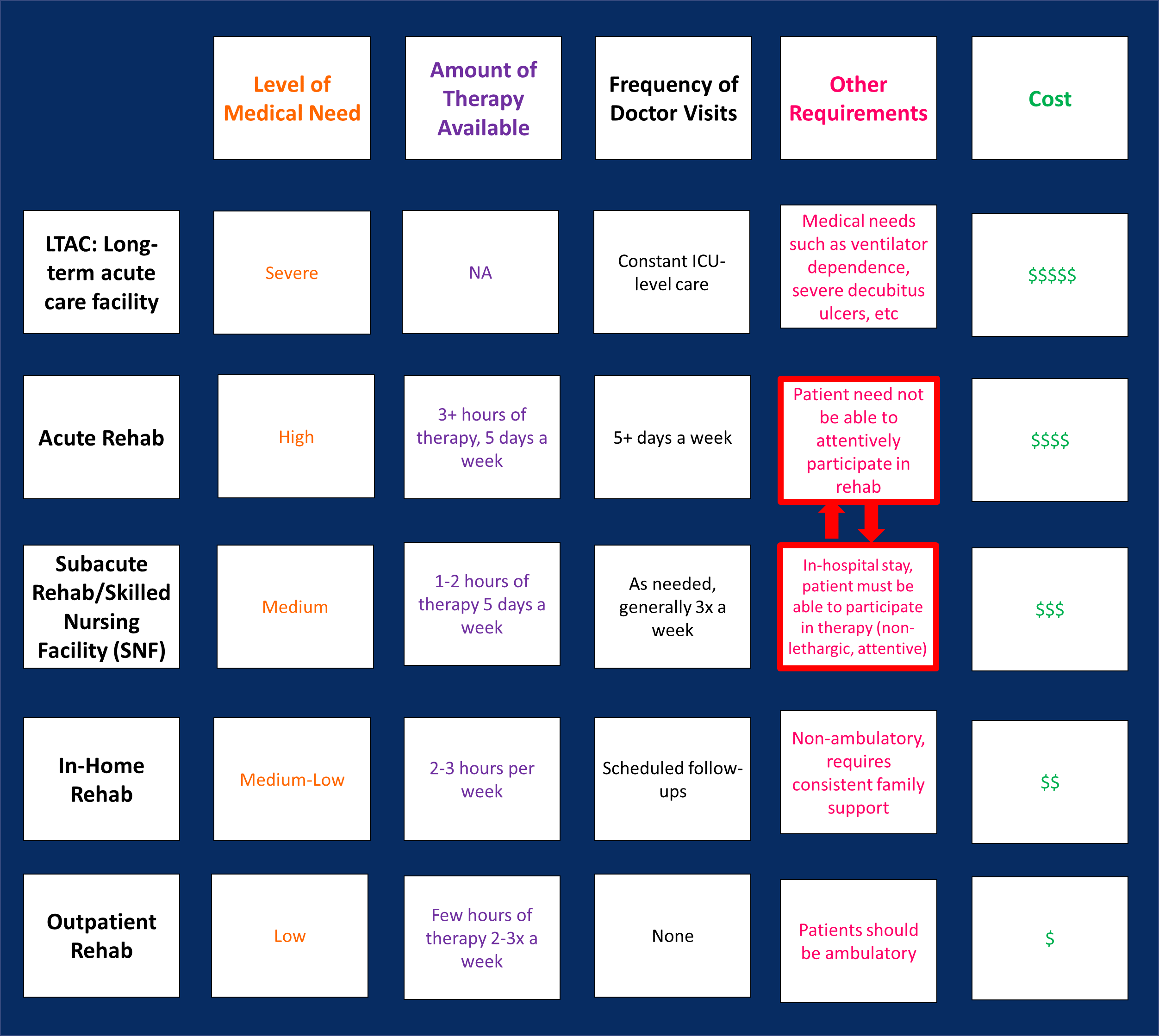


**Figure 1**: Mock-up of interactive activity students complete on simulated “Day 3”. a) Activity set-up. This is the “blank table” you will provide students with at the start of this portion of simulated “Day 3.” The table’s column and row headers should be arranged as above, top left. b) At the start of the activity, students will begin to affix cards to the felt using Velcro. c) Once students finish placing the cards where they feel they should go, the facilitator corrects any errors by switching cards as needed, using errors as teaching points. d) A visual of the finished interactive table. This table is re-produced on the student handouts.

**Level of Medical Needs**

Severe

High

Medium

**Outpatient Rehab**

Medium-Low

**Subacute Rehab/Skilled Nursing Facility** (SNF)

**LTAC**

(Long-term acute care)

**In-Home Rehab**

**Acute Rehab**

$

**Amount of Therapy Available**

NA

3+ Hours of Therapy, 5 days a week

1-2 Hours of Therapy, 5 days a week

Low

2-3 Hours per week

**Frequency of Doctor Visits**

Constant ICU-level care

5+ days a week

As needed, generally 3x a week

Few hours of therapy, 2-3x a week

Scheduled Follow-Ups

**Other Requirements**

Medical needs such as ventilator dependence, severe decubitus ulcers, etc

In-hospital stay, patient must be able to participate in therapy (non-lethargic, attentive)

Patient need not be able to attentively participate in rehab

None

Non-ambulatory, requires consistent family support

**Cost**

$$$$$

$$$$

$$$

Patients should be ambulatory

$$
