# Supplementary material for: An Interactive Approach to Teaching the Clinical Applications of Autonomy and Justice in the Context of Discharge Decision-Making
Source: MedEdPORTAL. 2020 Oct 16;16:10992. doi: 10.15766/mep_2374-8265.10992 (PMC7566224; doi:10.15766/mep_2374-8265.10992)
Supplement: Supplementary file 1 — Facilitator Guide.docxInstructions for Creating Interactive Table.docxStudent Handout.docxPretest.docxPosttest and Feedback Form.docx [file mep_2374-8265.10992-s001.zip › C. Student Handout.docx]

**Healthcare Decision-Making in the Context of Stroke: Student Handout**

1. **Hierarchy for surrogate decision-making in Ohio. Please refer to the American Bar Association for guidelines pertinent to your state.** https://www.americanbar.org/content/dam/aba/administrative/law_aging/2014_default_surrogate_consent_statutes.authcheckdam.pdf.

Spouse

Adult Child

Parents

Adult Sibling

Nearest Adult Relative

**Table comparing post-stroke rehabilitative facilities**

|  | **Level of Medical Needs** | **Amount of Therapy Available** | **Frequency of Doctor visits** | **Other requirements** | **Cost** |
| --- | --- | --- | --- | --- | --- |
| **LTAC (long-term acute care facility)** | Severe | NA | Constant ICU- level care | Medical needs such as ventilator dependence, severe decubitus ulcers, etc | $$$$$ |
| **Acute Rehab** | High | 3+ hours of therapy, 5 days a week | 5+ days a week | In-hospital stay, patient must be able to participate in therapy (non-lethargic, attentive) | $$$$ |
| **Subacute Rehab /Skilled Nursing Facility (SNF)** | Medium | 1-2 hours of therapy 5 days a week | As needed, generally 3 times a week | Patient need not be able to attentively participate in rehab | $$$ |
| **In-Home rehab** | Medium-Low | 2-3 hours per week | Scheduled follow-ups | Non-ambulatory, Requires consistent family support | $$ |
| **Outpatient Rehab** | Low | Few hours of therapy 2-3x a week | None | Patients should be ambulatory | $ |

**(Optional Resource) Link to Medicare resource allowing patients to compare inpatient rehab services:**

https://www.medicare.gov/inpatientrehabilitationfacilitycompare/
