# Supplementary material for: An Interactive Approach to Teaching the Clinical Applications of Autonomy and Justice in the Context of Discharge Decision-Making
Source: MedEdPORTAL. 2020 Oct 16;16:10992. doi: 10.15766/mep_2374-8265.10992 (PMC7566224; doi:10.15766/mep_2374-8265.10992)
Supplement: Supplementary file 1 — Facilitator Guide.docxInstructions for Creating Interactive Table.docxStudent Handout.docxPretest.docxPosttest and Feedback Form.docx [file mep_2374-8265.10992-s001.zip › E. Posttest and Feedback Form.docx]

**Post Activity Assessment and Feedback Form**

Facilitator note: Question 5 on this assessment pertains specifically to the surrogate hierarchy within Ohio. Please edit the appropriate responses based on the legal requirements in your own state, which can be found at https://www.americanbar.org/content/dam/aba/administrative/law_aging/2014_default_surrogate_consent_statutes.authcheckdam.pdf.

Name: ___________________________________________ Date: ______________________________

1. You have a 70 year old patient who has lost the ability to move the left side of her body as the result of a stroke. She is still able to physically feed herself, but at times forgets to feed herself or drink water. She has family in the area: an adult son who works as a police officer. She is able to tolerate 2 hours of therapy a day. Which is the appropriate discharge option for this patient?
   1. Long-term acute care (LTAC)
   2. Subacute/Skilled Nursing Facility (SNF)
   3. Outpatient Rehab
   4. Acute Rehab
   5. In-home rehab
   6. No rehab necessary
2. You have a 57 year old patient who ambulates with a cane at baseline that suffered an ACA stroke. Her family has noticed that his personality has shifted dramatically—although family members describe her as quiet and reserved prior to her stroke, in the hospital she is talkative, outgoing, and outspoken about her care. She initially resists treatment, but with repeated insistence ultimately complies with care. She is able to ambulate with a cane. Which is the appropriate discharge option for this patient?
   1. Long-term acute care (LTAC)
   2. Subacute/Skilled Nursing Facility (SNF)
   3. Outpatient Rehab
   4. Acute Rehab
   5. In-home rehab
   6. No rehab necessary
3. You have a 64 year old patient preparing for discharge after suffering an MCA stroke. Over the course of his 1 month long hospital stay, a PEG and trach tube are placed. His right upper extremity is very weak and he is unable to walk, but is attentive and actively participates in physical therapy. He can tolerate up to 3 hours of therapy a day. Which is the appropriate discharge option for this patient?
   1. Long-term acute care (LTAC)
   2. Subacute/Skilled Nursing Facility (SNF)
   3. Outpatient Rehab
   4. Acute Rehab
   5. In-home rehab
   6. No rehab necessary
4. What is the difference between competence and capacity?
   1. In the hospital setting, there is no difference
   2. Competence is a legal definition and capacity a medical definition
   3. Capacity is a legal definition and competence a medical definition
5. Assume you have an adult patient in Ohio who needs a surrogate decision-maker. Number the following in the order you would approach them to be a surrogate for this patient, with 1 being the first person you would contact.

___ Adult Child

___ Spouse

___ Parent

___ Cousin

___ Adult Sibling

**Feedback**

Rate the degree to which you agree or disagree with the following statements:

This activity was relevant to my clerkship education

Strongly disagree Somewhat disagree Neutral Somewhat Agree Strongly Agree

This activity presented new information:

Strongly disagree Somewhat disagree Neutral Somewhat Agree Strongly Agree

I learned something useful from this activity

Strongly disagree Somewhat disagree Neutral Somewhat Agree Strongly Agree

This activity achieved its stated goals (reproduced below)

Strongly disagree Somewhat disagree Neutral Somewhat Agree Strongly Agree

By the end of this activity:

1. Students will be able to list some ways in which stroke affects competence, and patients’ autonomy as a result.
2. Students will be able to list appropriate surrogate decision-makers for patients judged legally incompetent, and discuss how surrogates can be used to promote patient autonomy in proxy.
3. Students will be able to discuss the different levels of rehabilitative care, and how recommendations for placement determined as an extension of the ethical principle of justice

The overall quality of this activity was:

Poor Fair Average Good Excellent

Comments? Areas of strength or improvement?
