# Supplementary material for: An Interactive Approach to Teaching the Clinical Applications of Autonomy and Justice in the Context of Discharge Decision-Making
Source: MedEdPORTAL. 2020 Oct 16;16:10992. doi: 10.15766/mep_2374-8265.10992 (PMC7566224; doi:10.15766/mep_2374-8265.10992)
Supplement: Supplementary file 1 — Facilitator Guide.docxInstructions for Creating Interactive Table.docxStudent Handout.docxPretest.docxPosttest and Feedback Form.docx [file mep_2374-8265.10992-s001.zip › _Educational_Summary_Report_10992.pdf]

MedEdPORTAL® The AAMC Journal of Teaching and Learning Resources

Original Publication Open Access

An Interactive Approach to Teaching the Clinical Applications of Autonomy and Justice in the Context of Discharge Decision-Making

Divya Palanisamy*, Wei Xiong, MD

*Corresponding Author: dpalanis@bidmc.harvard.edu

Abstract

Introduction: Students are taught the basics of medical ethics during their pre-clinical education, but need additional instruction on how to apply these principles to patient situations they may encounter during their clinical rotations. The ethical principles of autonomy and justice become especially pertinent to patient care in the setting of discharge decision-making. Third-year medical students would therefore benefit from an interactive educational activity that allows them to practice applying these principles within the context of discharge decision-making. **Methods:** This session was designed for third-year medical students completing their required rotation in neurology. Students participated in a 1-hour, facilitator-led, interactive, small-group, ethics-based activity meant to simulate the typical 4-day post-stroke hospital stay. Learning objectives for the activity were to enhance students' understanding of the principles of autonomy, justice, competence, and capacity. Students were given pretest to gauge prior knowledge of activity learning objectives; their knowledge was again assessed afterwards, and they were surveyed on the usefulness and value of the activity. **Results:** Twenty-three third-year medical students completed the activity over three sessions. The average improvement between pre- and posttest score was 40%. Lastly, on the qualitative feedback form, a majority of students strongly agreed that the activity was useful and presented new information, with 18 of 23 students giving the activity the highest possible rating of *excellent*. **Discussion:** A large majority of the students found the activity to be valuable, and the activity was shown to be effective at improving students' knowledge of a key aspect of successful medical practice.

Keywords
Discharge Planning, Ethics, Competence and Capacity, Autonomy, Justice, Clinical Reasoning/Diagnostic Reasoning, Ethics/Bioethics, Feedback, Quality Improvement/Patient Safety, Neurology, Clinical Teaching/Bedside Teaching, Multimedia, Qualitative Research, Quantitative Research

Educational Objectives

By the end of this activity, students will be able to:

1. List some ways in which stroke affects competence, and patients' autonomy as a result.
2. List appropriate surrogate decision-makers for patients judged legally incompetent, and discuss how surrogates can be used to promote patient autonomy in proxy.
3. Discuss the different levels of rehabilitative care, and how recommendations for placement determined as an extension of the ethical principle of justice.

Citation:
Palanisamy D, Xiong W. An interactive approach to teaching the clinical applications of autonomy and justice in the context of discharge decision-making. *MedEdPORTAL*. 2020;16:10992.
https://doi.org/10.15766/mep_2374-8265.10992

Introduction

The importance of teaching ethics as a part of medical education has been formally recognized since 1985, and has been included as a core standard of accreditation for many years by the Liaison Committee on Medical Education (LCME).[1,2] However, there is still a need for additional coverage of these topics: in one survey of 336 medical students and residents, only 18% of respondents felt the professionalism and ethics preparation they received was sufficient.[3] During preclinical years, most students are taught the four basic principles of beneficence, nonmaleficence, autonomy, and justice, and then are often given simplified mock patient scenarios using which they can practice applying each principle in isolation. In their subsequent clinical training, it is often left to students to glean how the different ethical principles are applied in daily patient care decisions.[4] However, when left to apply these principles independently, they lose the opportunity to benefit from the expert guidance of practicing physicians, who can

Copyright © 2020 Palanisamy and Xiong. This is an open-access publication distributed under the terms of the Creative Commons Attribution license.

speak to how these principles apply in every patient scenario, rather than those most obviously ethical. There is thus a need for practitioner-led, contextualized teaching of professional ethics in clinical medical education.

We specifically hypothesized that a tactile, interactive clerkship activity would allow for medical ethics to be taught in an appropriately contextualized manner that would foster deeper understanding of previously learned material and facilitate professional development. There is ample evidence that medical students would benefit from additional instruction in medical ethics, and we intended for this project to be one example of how that might be achieved with minimal disruption to preexisting clerkship requirements. Although many medical educators across disciplines have recognized the need for instruction in ethics that is integrated in clinical training, there are few examples of instruction specifically targeted towards third-year medical students.[5-8] Furthermore, many of these existing examples limited the scope of the activity to one specific ethical situation (such as genomics, or the approach to Jehovah's Witness patients) with limited generalizability to other patients or other specialties.[5-8] In contrast, the activity we designed was broad in scope: while it was framed using the example of a poststroke patient, this activity contextualized the ethical principles of autonomy and justice within the framework of discharge decision-making, which is broadly applicable across specialties. In addition, to our knowledge this was the first implementation of an interactive activity to be used to teach discharge decision-making. Our work supplements existing literature as an example of how students can be taught to recognize ethical considerations in patients who initially may not seem ethically complicated.

In this activity, we contextualized the core ethical principles of autonomy and justice within the practical framework of assessing patient capacity and discharge decision-making for a patient recovering from acute neurological impairment. Since acute neurological impairment can quickly alter both mental and physical functional ability, medical providers are often the first to discuss these topics with patients and their families. The third-year neurology clerkship thus presented an ideal situation in which to add to, and reinforce, students' preclinical ethics education. The question of how and when to discharge a patient with neurologic impairment to a particular rehabilitation facility is a key feature of a patient's medical care, and as it relates to resource allocation, is a practical application of the ethical principle of justice. Likewise, when patients lose capacity and require a surrogate decision-maker, it is a practical application of the ethical principle of autonomy. However, medical students are often not given detailed foundational information on what discharge planning and capacity assessment entail, and are thus unable to fully participate in or learn from these key conversations. Because they are lacking the foundational knowledge, they are unable to recognize and take advantage of the learning opportunities they are presented with every day. We further hypothesized that an educational activity that allowed students to practice applying ethical principles specifically as they related to discharge decision-making for stroke patients would be particularly salient to third-year medical students.

Methods

We designed an ethics-based activity meant to simulate the typical poststroke hospital stay. Learning objectives for the activity were designed to teach students about the concept of competence versus capacity, the state-specific legal hierarchy for surrogate decision-makers, and the options available for poststroke rehabilitative care, as well as the requirements for placement in each of those options.[9-11] This activity was designed specifically for third-year medical students. All students who completed their third-year core clerkship in neurology at the academic hospital in which this study was conducted during the 3-month study duration were included for participation. This lesson was done for students at the end of their 1-month core clerkship in neurology. We asked students to first complete a brief five-question pretest to gauge prior knowledge of activity learning objectives. The students then participated in a 1-hour, facilitator-led, interactive, small-group session. Following the activity, we once again assessed their knowledge with a five-question quiz. We also surveyed participating students on the usefulness and value of the activity. Ideally, the activity would be facilitated by a medical provider with experience leading discussions of capacity and discharge planning, within the context of neurological impairment, who can in turn share their experience with students and answer any questions they may have. Each iteration of the activity we presented was led by one or both authors of this study. Our project qualified for institutional review board exemption because it was conducted in established educational settings and involved normal educational practices and survey procedures.

Due to the prevalence and predictability of the disease and its management, we chose ischemic stroke as the basis for the activity. According to the American Stroke Association, the average hospital stay following an ischemic stroke was 4 days.[12] Therefore, the activity was split into four parts, simulating 4 hospital days.

Copyright © 2020 Palanisamy and Xiong. This is an open-access publication distributed under the terms of the Creative Commons Attribution license.

Simulated Day 1 (10 Minutes)

The educational focus for this simulated day was the difference between competence and capacity. We asked students to place themselves in the shoes of the stroke patient, and split each group of students into two teams as outlined in the facilitator guide (Appendix A). During this simulated day, the facilitator asked each team to consider what capacity-altering deficits they would likely have after an ischemic stroke in either an anterior cerebral artery territory or a middle cerebral artery territory lesion. The students discussed, first in their teams and then as a large group. The facilitator used the discussion to teach students about the difference between competence and capacity, and then further asked students to generate strategies they could use in the inpatient setting to assess a patient's decision-making capacity.

Simulated Day 2 (10 Minutes)

The educational focus for this day of the simulation was to teach students the hierarchy of surrogate decision makers. During this day, students considered the perspective of the family. Students were told that their stroke patient was determined not to have capacity, and then given a list of relations (i.e., spouse, sister, cousin, parents, etc.) to rank as surrogate decision makers, with information from the American Bar Association on surrogate consent statutes serving as reference.[5] The students initially worked in their separate teams, and later the two teams compared their ranked lists. The activity was set up such that there would be some relations whom the law determined to be higher up the list than those who may be closer—for example, the students were given the choice of a sibling who lived far away and a cousin who lived much closer and was in better contact with the patient. The students were encouraged to discuss how this might affect patient care.

Simulated Day 3 (20 Minutes)

The educational focus for this day was to give students the foundational knowledge necessary for students to understand discussions of discharge decision-making through a tactile interactive activity. Instructions for how to create the large, interactive table necessary for this segment of the activity were listed in Appendix B. For this activity, students continued to work in their two teams. The facilitator began by pulling out a large blank table (Appendix B). The backdrop was made of felt, and each of the white boxes below was meant to represent an index card that could be affixed to the felt using Velcro. The table entries were given to students in both teams in a random order, such that each team had half of the entries needed to complete the table. Each of the entries they were given were also printed on small index cards, each with Velcro on the back. They began to work within their teams to fill in the table, using the Velcro to affix table entries in what they believed to be the appropriate row and column (Appendix B). Once the students had completed the table to the best of their ability, the facilitator used the key provided in the facilitator guide to review their work. They then used any mistakes in the table as teaching points (Appendix B) to conclude the activity.

Simulated Day 4 (5 Minutes)

The educational focus of this day was to leave students with practical resources they could in turn pass along to their patients, and to leave flexible time for facilitators to answer any questions that came along earlier in the activity. At the end of the activity, the facilitator gave each student a handout (Appendix C) with some key points from the activity, including the hierarchy for surrogate decision makers in Ohio, and a completed version of the interactive table they constructed on day 3.

Assessment

We assessed students using the same five-question assessment before and after the activity (Appendices D and E). Four of the five questions were multiple choice. Since one primary goal was to evaluate the effectiveness of the tactile, interactive activity, three of the five questions presented a description of a patient and asked students to choose the appropriate discharge option. The fourth question, also multiple choice, tested students' ability to correctly distinguish between competence and capacity. The fifth and final question gave students a list of possible surrogate decision makers and asked them to order the surrogates from whom they would contact first to whom they would contact last.

At the end of the activity, we also asked students to provide qualitative feedback on the activity. We asked each student to give a quality rating for the overall activity, choosing from options *poor*, *fair*, *average*, *good*, or *excellent*. We also included space for students to comment on areas of strength or improvement for the activity.

Results

The success of this activity was assessed with both quantitative and qualitative measures. Twenty-three students completed the activity over three separate sessions. Students' performance on the pretest was compared to their performance on the posttest in Figure 1. Of the students, 22 of 23 showed an improvement of at least 1 point between the pre- and postest, each of which were out of 5 points. One student's pre- and posttest score remained the same. The average improvement between pretest

Copyright © 2020 Palanisamy and Xiong. This is an open-access publication distributed under the terms of the Creative Commons Attribution license.

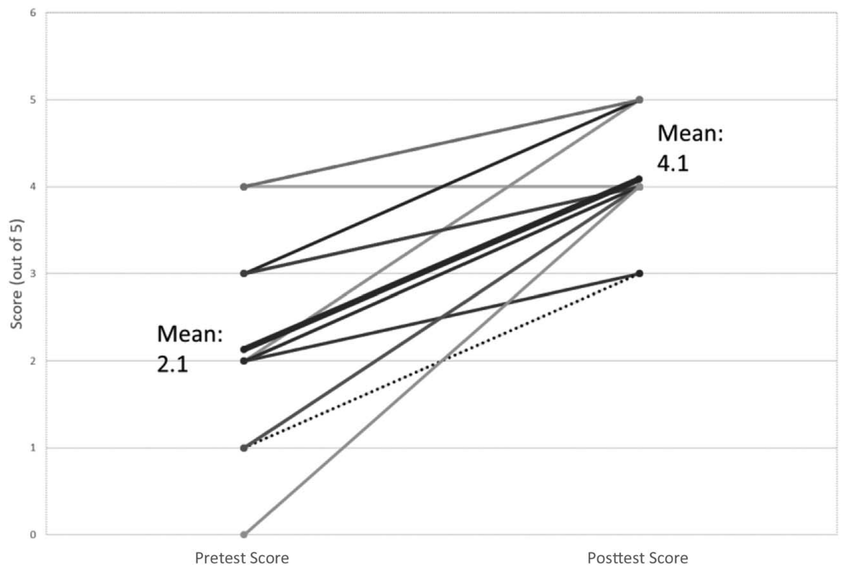

Figure 1. Pre- and posttest performance. Students scored 1 point for each correct answer (max 5 points). Mean pre- and posttest scores are shown for all students (n = 23), as well as individual pre- and posttest score changes for each participant.

and posttest score was 2 points. The Table provides a breakdown of correct responses by learning objective.

Results from the qualitative feedback form were shown in Figure 2. Overall, 18 of 23 (78%) students gave the activity the highest possible rating of *excellent*, with the remaining 5 of 23 (22%) giving it the next highest rating of *good*. Students commonly commented on the novelty and usefulness of the activity (e.g., "Very unique and useful. I've never been taught about this before but now I will understand dispo better."). Students also commonly commented specifically that they enjoyed the interactive component (e.g., "I enjoyed the activity pinning the labels to the felt board—more interactive than being lectured at.").

Constructive feedback from participants included comments that the information would have been more helpful earlier in the rotation, prior to clinical experience, such as, "Maybe do at beginning of neuro rotation so you know this info early on and can use it and apply it on the wards."

Discussion

The goal of this activity was to reinforce and add to students' practical knowledge of medical ethics as it applies to assessing patient capacity and planning for discharge within the context of acute neurological impairment. Although this activity did not involve direct patient contact, discussion questions were framed such that they required students to reflect on the experiences they have had in clinical settings, thus bridging the gap between preclinical theory and hospital experience. The activity met its goals—students reacted positively to the interactive activity qualitatively, and pre- and posttest scores demonstrated their factual knowledge of activity goals improved as well. The novel use of an interactive method of teaching was shown to be effective in achieving our educational objectives.

The strength of our analysis as to the utility of this activity was primarily limited by the fact that assessments were presented immediately following the activity, and we were thus unable to gauge students' long-term retention of material. It was

Table. Percentage of Correct Responses by Learning Objective (n = 23)

Learning Objective and Pertinent Assessment Questions	Correct Responses (%)		
	Pretest	Posttest	*P*
1. Students will learn the options available for post-stroke rehabilitative care as well as the requirements for placement in each of those options (Q1-Q3).	30	71	<.001
2. Students will learn the state-specific hierarchy for surrogate decision-makers, and learn to distinguish competence from capacity (Q4 and Q5).	63	98	<.001

Copyright © 2020 Palanisamy and Xiong. This is an open-access publication distributed under the terms of the Creative Commons Attribution license.

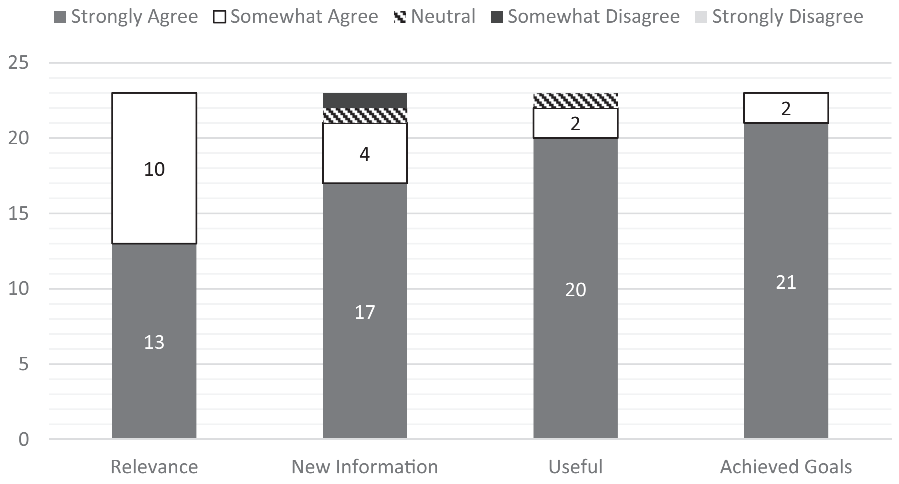

Figure 2. Student posttest ratings of the activity ($n = 23$).

interesting to find that a student in our cohort, who had been previously exposed to this material, did not feel that the quality of the activity suffered because the material presented was not new. Furthermore, although this student's subjective ratings of the activity's relevance and utility were lower compared to others' ratings, objectively the student's knowledge improved, highlighting the importance of repetition.

This activity was designed as an introductory session, and although the students who participated were at least midway through their clerkship year, all but one had not been exposed this material. This speaks to the need for formal education on the topics of discharge decision-making. This activity could thus be modified to suit patients with complaints pertinent to all specialties. It could then be introduced to students during whichever rotation they experience as their first clerkship. This would standardize when students are exposed to this material during their clerkship rotations, and the early introduction would allow for them to reinforce what they learn as they gain more clinical experience. It would also allow them to compare and contrast the ways in which these principles are applied as they experience the different clerkship rotations.

The limitations of this study primarily involved its scope. The short duration of the study and small sample size both limited the generalizability of our results. Furthermore, although we hoped that versions of this activity could be successfully implemented in other core clerkships, we have only demonstrated its success in the setting of the neurology clerkship. In this study, the facilitator in each session had also participated in developing the lesson plan. We were thus unable to say if a naïve facilitator given only the facilitator guide would be able to reproduce these results. The facilitators were also given the freedom to entertain questions from students that may not be included in the facilitator guide. The student experience session-to-session therefore was not identical, and our pre- and posttest assessments were not detailed enough to assess if these deviations had any effect on qualitative or quantitative performance.

In summary, this session addressed the need for ethical education that is longitudinal and appropriately contextualized within the practical frameworks they will need to use while on the wards. We presented it within the context of acute neurological impairment, but discussions of capacity and discharge planning are broadly applicable to all specialties. Based on the exceedingly positive qualitative and quantitative feedback from participants, we felt that our interactive activity was valuable to students and met its intended goals of reinforcing ethical principles of justice and autonomy as they apply to assessing capacity and planning for discharge.

Appendices

A. Facilitator Guide.docx

B. Instructions for Creating Interactive Table.docx

C. Student Handout.docx

D. Pretest.docx

E. Posttest and Feedback Form.docx

All appendices are peer reviewed as integral parts of the Original Publication.

Copyright © 2020 Palanisamy and Xiong. This is an open-access publication distributed under the terms of the Creative Commons Attribution license.

Divya Palanisamy: Fourth-Year Medical Student, Case Western Reserve University School of Medicine

Wei Xiong, MD: Assistant Dean for Clerkship Education, Case Western Reserve University School of Medicine; Core Clerkship Director and Assistant Professor, Department of Neurology, University Hospitals Cleveland Medical Center

Disclosures
None to report.

Funding/Support
None to report.

Prior Presentations
Palanisamy D, Xiong W. An ethics-based approach to teaching discharge decision-making in the context of neurological impairment. Poster presented at: Medical Education Retreat; 2019; Cleveland, OH.

Ethical Approval
Reported as not applicable.

References

1. Culver CM, Clouser KD, Gert B, et al. Basic curricular goals in medical ethics. *N Engl J Med*. 1985;312(4):253-256. https://doi.org/10.1056/NEJM198501243120430
2. Liaison Committee on Medical Education. *Functions and Structure of a Medical School: Standards for Accreditation of Medical Education Programs Leading to the MD Degree*. Liaison Committee on Medical Education. Published 2016. Accessed August 21, 2020. https://www.aamc.org/services/first-for-financial-aid-officers/lcme-accreditation
3. Roberts LW, Hammond KAG, Geppert CMA, Warner TD. The positive role of professionalism and ethics training in medical education: a comparison of medical student and resident perspectives. *Acad Psychiatry*. 2004;28(3):170-182. https://doi.org/10.1176/appi.ap.28.3.170
4. Carrese JA, Malek J, Watson K, et al. The essential role of medical ethics education in achieving professionalism: the Romanell Report. *Acad Med*. 2015;90(6):744-752. https://doi.org/10.1097/ACM.0000000000000715
5. Spike J. “Dax’s Case”: autonomy and paternalism. *MedEdPORTAL*. 2008;4:785. https://doi.org/10.15766/mep_2374-8265.785
6. Dasgupta S. Medical genetics ethics case collection: discussion materials for medical students in the genomic era. *MedEdPORTAL*. 2017;13:10562. https://doi.org/10.15766/mep_2374-8265.10562
7. Dingle A, DeJong S, Madaan V, Ascherman L. Teaching ethics in child and adolescent psychiatry: vignette-based curriculum. *MedEdPORTAL*. 2016;12:10418. https://doi.org/10.15766/mep_2374-8265.10418
8. Malkin M, Lenart J, Walsh CA, Woodfin M, Vadi M. Transfusion ethics in a pediatric Jehovah’s Witness trauma patient: simulation case. *MedEdPORTAL*. 2016;12:10450. https://doi.org/10.15766/mep_2374-8265.10450
9. Default surrogate consent statutes. American Bar Association. September 2019. Accessed September 2020. https://www americanbar.org/content/dam/aba/administrative/law_aging/2019-sept-default-surrogate-consent-statutes.pdf
10. Stroke: rehabilitation services. Cleveland Clinic. Updated March 1, 2016. Accessed December 2018. https://my.clevelandclinic.org/health/articles/5600-stroke-rehabilitation-services
11. Winstein CJ, Stein J, Arena R, et al. Guidelines for adult stroke rehabilitation and recovery: a guideline for healthcare professionals from the American Heart Association/American Stroke Association. *Stroke*. 2016;47(6):e98-e169. https://doi.org/10.1161/STR.0000000000000098
12. Caswell J. What happens next? Making the best decisions at discharge after stroke. *Stroke Connect*. Summer 2017. https://strokeconnection.strokeassociation.org/Summer-2017/What-Happens-Next-Making-the-Best-Decisions-at-Discharge-After-Stroke/

Received: August 7, 2019
Accepted: April 28, 2020
Published: October 16, 2020

Copyright © 2020 Palanisamy and Xiong. This is an open-access publication distributed under the terms of the Creative Commons Attribution license.
